# Regulated Iron Siderophore Production of the Halophilic Archaeon *Haloferax volcanii*

**DOI:** 10.3390/biom10071072

**Published:** 2020-07-17

**Authors:** Natalie Niessen, Jörg Soppa

**Affiliations:** 1 Institute for Molecular Biosciences, Goethe-University, Biocentre, Max-von-Laue-str. 9, D-60439 Frankfurt, Germany; nmiessen@gmail.com; 2 Campus Callaghan, Faculty of Health and Medicine, School of Medicine and Public Health, Hunter Medical Research Institute, University of Newcastle, Newcastle, NSW 2308, Australia

**Keywords:** Archaea, *Haloferax volcanii*, siderophore, hydroxamate, deletion mutant, O-CAS assay, iron starvation, Schizokinen

## Abstract

Iron is part of many redox and other enzymes and, thus, it is essential for all living beings. Many oxic environments have extremely low concentrations of free iron. Therefore, many prokaryotic species evolved siderophores, i.e., small organic molecules that complex Fe^3+^ with very high affinity. Siderophores of bacteria are intensely studied, in contrast to those of archaea. The haloarchaeon *Haloferax volcanii* contains a gene cluster that putatively encodes siderophore biosynthesis genes, including four iron uptake chelate (*iuc*) genes. Underscoring this hypothesis, Northern blot analyses revealed that a hexacistronic transcript is generated that is highly induced under iron starvation. A quadruple *iuc* deletion mutant was generated, which had a growth defect solely at very low concentrations of Fe^3+^, not Fe^2+^. Two experimental approaches showed that the wild type produced and exported an Fe^3+^-specific siderophore under low iron concentrations, in contrast to the *iuc* deletion mutant. Bioinformatic analyses revealed that haloarchaea obtained the gene cluster by lateral transfer from bacteria and enabled the prediction of enzymatic functions of all six gene products. Notably, a biosynthetic pathway is proposed that starts with aspartic acid, uses several group donors and citrate, and leads to the hydroxamate siderophore Schizokinen.

## 1. Introduction

Iron is an essential element for all living beings. It is, for example, a constitutive part of ion sulfur clusters, which are involved in the redox chemistry of many enzymes. These include membrane-bound enzymes of aerobic and anaerobic electron transport chains; thus, iron is indispensable for energy metabolism. Due to the low solubility of Fe^3+^ salts, free iron occurs at very low concentrations and is growth-limiting in many oxic environments. Therefore, many aerobic microorganisms developed efficient iron chelatores, small organic molecules which are referred to as siderophores. They are typically produced only at low extracellular iron concentrations, are secreted, and bind Fe^3+^ with high affinity. When loaded with iron, they are taken up by high-affinity uptake systems, and iron is liberated in the cytoplasm. Based on their chemical structures, siderophores are classified into various types: catecholate, carboxylate, hydroxamate, phenolate, or mixed type. Because siderophores are exported to the environment, they can not only be used by the producing species, but can affect additional members of microbial communities and can lead to competitive or cooperative interactions [1]. Iron is also important for the interaction between pathogenic microorganisms and their eukaryotic hosts. Many hosts developed iron-binding proteins (e.g., ferritin, transferrin) to sequester iron, thereby limiting microbial growth. To overcome this hurdle, many pathogenic microorganisms produce siderophores with high Fe^3+^ affinity to guarantee a sufficient iron supply in spite of the low intra-host concentration and, thus, part of the pathogen–host interaction is a battle for iron [2].

The biosynthesis of siderophores, as well as its regulation, was intensely studied in various groups of bacteria [3,4,5]. However, nearly nothing is known about siderophores of archaea. Only about five publications on siderophores and Archaea exist, not always reporting the ability to produce siderophores. Very recently, it was described that the ammonia-oxidizing archaeon *Nitrosopumilus maritimus* is unable to produce its own siderophore, but that it can use an exogenous siderophore for iron uptake [6]. Earlier, it was described that five of nine Indian isolates of seven different genera of halophilic Archaea are capable of siderophore production [7]. This publication, as well as an additional publication, reported that *Halobacterium salinarum* does not produce a siderophore [7,8]. However, several different exogenous siderophores promote the growth of *H. salinarum*, indicating that it can use them for Fe^3+^ acquisition [8]. The genome of *Haloferax volcanii* contains four genes that are annotated as *iuc* (iron uptake chelate) genes based on the similarity of the encoded proteins to bacterial siderophore biosynthesis proteins [9] (www.halolex.mpg.de). Comparison of the transcriptomes of *H. volcanii* cultures grown via anaerobic nitrate respiration in comparison to aerobic respiration showed that the *iuc* transcripts are induced under aerobic conditions, which would be consistent with their putative function as siderophore biosynthesis enzymes (unpublished data). The aim of the study presented here was to clarify the biological role of the putative *iuc* genes, specifically whether or not they are indeed involved in siderophore biosynthesis. An *iuc* gene deletion mutant was generated and compared to the wild type with regard to growth and siderophore biosynthesis. In addition, differential expression of the *iuc* genes was studied. It could in fact be verified that the *iuc* genes are essential for siderophore production and, thus, *H. volcanii* is the first archaeal species for which siderophore biosynthesis genes are experimentally validated.

## 2. Materials and Methods

### 2.1. Strains and Culture Conditions

The *H. volcanii* strain H26 [10] was used as a parent strain for deletion mutant generation. It is herein called “wild type”, because it has the wild-type genomic organization for the genes of interest of this study (HVO_B0041 to HVO_B0046). *H. volcanii* was grown in complex medium under optimal conditions (42 °C, 250 rpm, 30 mL of culture in 100-mL Erlenmeyer flasks).

### 2.2. Transcript Analysis

Recently, the transcriptome of *H. volcanii* was analyzed with dRNA-Seq to identify transcription start sites (TSS) [11] and with RNA-Seq to characterize transcript lengths and operon structure [12]. The results were visualized with the Integrated Genome Browser [13] to get a first impression of the transcripts of the HVO_B0041 to HVO_B0046 gene cluster.

For further analyses, Northern blot analyses with digoxigenin-labeled probes were performed as previously described [12]. The Northern blots were scanned, and the signals were quantified using the software ImageJ (imagej.nih.gov; version 1.52; National Institute of Health, Bethesda, MD, USA). Identical areas were used for signal quantification and background subtraction. Three biological replicates were performed, and average values and their standard deviations were calculated.

### 2.3. Construction of a Deletion Strain

An iucABDC deletion strain (HVO_B0041 to HVO_B0044) was generated using the previously described pop-in/pop-out method [10,14]. The sequences of the oligonucleotides are listed in Table S1 (Supplementary Materials). As *H. volcanii* is highly polyploid, great care was taken to verify the homozygosity of the mutant, i.e., both Southern blot analysis and multi-cycle PCR analysis were performed for deletion mutant verification.

### 2.4. Analysis of Fe-Dependent Growth

The analyses of iron-dependent growth were performed in synthetic medium [15] with 0.5% (*w*/*v*) glucose as the sole carbon and energy source. Extra pure NaCl (>99.999%; Fluka, Neu-Ulm, Germany) was used to avoid the introduction of trace amounts of iron into the medium. FeCl_2_ and FeCl_3_ were added at concentrations indicated in Section 3. For the analyses of growth curves, cultures were grown in microtiter plates as previously described [15].

### 2.5. Siderophore Assays

Two distinct assays were performed for the analysis of siderophore production.

(1) FeCl_3_ assay [16]. Cultures were grown in liquid synthetic medium with extra pure NaCl and 0.5% (*w*/*v*) glucose. Precultures were grown in the absence of FeCl_3_ overnight. Test cultures were grown in the presence of 20 µM FeCl_3_. After five days of incubation, cells were pelleted and the supernatant was used for the assay. Then, 0.5 mL of supernatant was added to 2.5 mL of FeCl_3_ solution (2% (*w*/*v*)). Generation of a brown color was indicative for the presence of a siderophore.
(2) The O-CAS assay (overlay chromeazurol S assay) [17] was developed to enable the analyses of siderophore production for species that are sensitive to the CAS reagent. It consists of a prior growth phase and a subsequent analysis with the CAS reagent. Precultures were grown in the absence of FeCl_3_ in liquid synthetic medium with extra pure NaCl and 0.5% (*w*/*v*) glucose. Cells numbers were counted using a Neubauer counting chamber, and suitable dilutions were generated to yield the cell numbers indicated in Section 3. Next, 5 µl of the dilutions were spotted on solid medium (extra pure NaCl, 0.5% glucose (*w*/*v*), 1% (*w*/*v*) agar) containing the indicated FeCl_3_ concentrations. The agar plates were incubated for four days at 42 °C to allow the formation of small colonies. The plates were overlaid with 1× CAS solution with 0.9% (*w*/*v*) agar and incubated at room temperature (10× CAS reagent: 0.1 mM FeCl_3_, 1 mM HCl, 2 mM HDTMA (Sigma-Aldrich, Taufkirchen, Germany; H52366; hexadecyltrimethylammonium bromide), 1 mM CAS = chromeazurol S, Sigma 72687). In the absence of a siderophore, a blue-green color develops in the plate. The presence of uncolored halos around colonies is indicative for the production and export of a FeCl_3_ siderophore.

### 2.6. Databases and Bioinformatic Analyses

The genome of *H. volcanii* was addressed using the website HaloLex (www.halolex.mpg.de) [18]. The curated database contains, e.g., features of the genes and proteins, and it allows downloading gene and protein sequences. In addition, the UniProt database was used (https://www.uniprot.org/uniprot/). The presence of known protein domains was analyzed at the InterPro database (https://www.ebi.ac.uk/interpro/protein/UniProt). The String database was used for the proposal of potential protein-protein-interaction networks (https://string-db.org/).

The search for homologous proteins was performed with BLASTp (Basic Local Alignment Search Tool) at the following website: https://www.ebi.ac.uk/Tools/sss/ncbiblast/. The UniProtKB taxonomic subsets “archaea”, “bacteria”, and “eukaryotes” were searched separately, and the 1000 most similar proteins were listed. Multiple sequence alignments were generated using ClustalOmega (https://www.ebi.ac.uk/Tools/msa/clustalo/).

## 3. Results

### 3.1. A Gene Cluster Possibly Involved in Siderophore Biosynthesis

**Figure 1:**
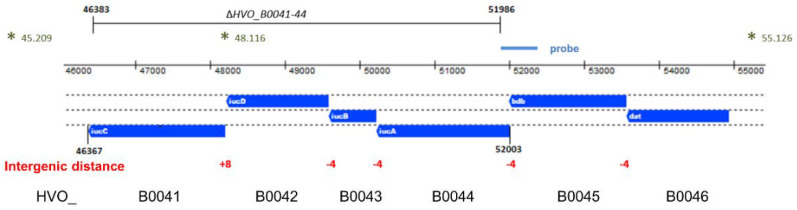
Schematic overview of the genomic region of the iron uptake chelate (*iuc*) gene cluster. The genes are shown in blue below the genomic coordinates. The intergenic distances between the genes are shown in red. The gene names are shown in white within the genes, and the HVO_ designations are shown at the bottom. The region that was deleted in the quadruple deletion mutant is indicated at the top. The asterisks indicate the restriction enzyme sites that were used for the Southern blot analysis; the genomic coordinates of the sites and the position of the probe are also indicated.

The genome of *H. volcanii* contains four genes that are annotated as *iuc* (iron uptake chelate) genes, which could possibly encode enzymes involved in siderophore biosynthesis. The respective genes form the *iucABDC* gene cluster (HVO_B0041 to HVO_B0044) and are localized on the reverse strand of the minor chromosome pHV3. The genomic organization is schematically shown in Figure 1. There are 4-nt overlaps of the gene pairs *iucAB* and *iucBD*. Recently, it was shown that gene pairs with 4-nt overlaps are typically indicative for translational coupling, meaning that translation of the downstream gene depends on translation of the upstream gene [19]. Therefore, the gene pairs *iucAB* and *iucBD* should be translationally coupled. The intergenic distance between *iucD* and *iucC* is only 8 nt and, thus, too small to contain a complete Shine Dalgarno motif. Therefore, in this case, translational coupling might also be predicted. Upstream of the four *iuc* genes are two further genes, which form two additional pairs of genes with 4-nt overlaps, i.e., *bdb-iucA* and *dat-bdb*, which might also be translationally coupled. The distance of the next gene, HVO_B0047, is 457 nt, making it too large to predict co-transcription. Taken together, the genes HVO_B0046 to HVO_B0041 form a cluster of six genes that might be translationally coupled throughout. However, this scenario would require transcription of the six genes into one polycistronic transcript. Therefore, transcript analyses were performed.

### 3.2. Transcript Analyses and Differential Expression

**Figure 2:**
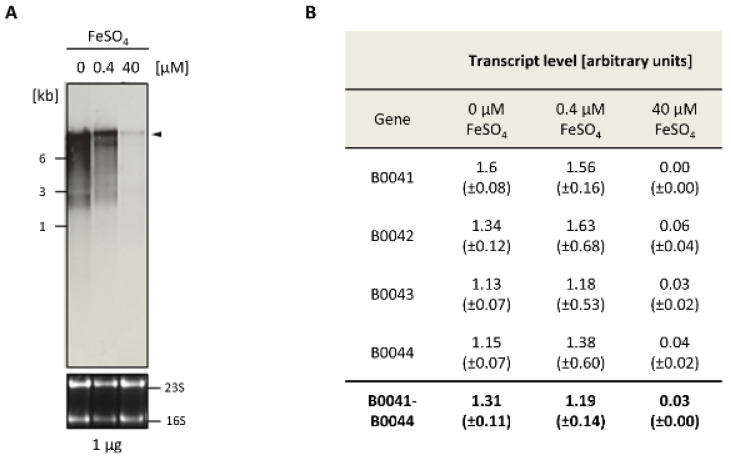
Differential expression of the *iuc* genes in the absence of iron and at two Fe^3+^ concentrations. (**A**) Northern blot analysis. The FeSO_4_ concentrations are indicated at the top. The upper part shows the signals of the Northern blot with a probe against *HVO_B0044*, while the lower part shows the stained RNA gel with the 16S and 23S ribosomal RNA (rRNA) bands. (**B**) Summary of the quantification of the messenger RNA (mRNA) levels with four probes that were specific for the four *iuc* genes. The Northern blots were scanned, and the signals were quantified with the software ImageJ (imageJ.nih.gov). Rectangular frames were used for signal quantification, and frames of the same size were used for background subtraction. Average values for three biological replicates and their standard deviations are shown. At the bottom, the averages for the four probes are shown.

Northern blot analyses were performed to analyze the expression of the *iuc* gene cluster. Cultures of the wild-type H26 were grown in the absence of FeCl_3_ and, respectively, in the presence of 0.4 µM and 40 µM FeCl_3_. Total RNA was isolated and Northern blots analyses were performed with four different probes that were specific for each of the four *iuc* genes. Figure 2A shows the analysis with the *iucA*-specific probe (HVO_B0044). At the concentration of 40 µm FeCl_3_, only trace amounts of a large transcript were observed, which was highly induced in cultures grown at the lower concentration of 0.4 µM FeCl_3_ and in the absence of iron. The size of the transcript was about 9000 nt, which fits to the size of a hexacistronic transcript of HVO_B0046–HVO_B0041 (about 8600 nt without UTRs), and which is much too large for a tetracistronic *iucABDC* transcript (about 5600 nt). In the fully induced state, small amounts of three smaller transcripts were visible, which might originate from partial transcription termination. However, notably, the major transcript is about 9000 nt in length and is much longer than an average transcript, which has a length of about 1000 nt [12]. One major large transcript of about 9000 nt was also observed with probes for the genes HVO_B0041, HVO_B0042, HVO_B0043, and HVO_B0046, underscoring co-transcription of the six genes HVO_B0046 to HVO_B0041 into one common hexacistronic transcript.

For the analysis of differential transcript level regulation, the amount of the polycistronic transcript was quantified with each of the four *iuc* probes. Figure 2B shows the individual values, as well as the average value obtained with the four probes. The level of the polycistronic transcript was on average about 44-fold higher in cells cultivated in the absence of FeCl_3_, compared to cells cultivated in the presence of 40 µM FeCl_3_. This result is perfectly compatible with the idea that the *iuc* gene cluster encodes enzymes for Fe^3+^ siderophore biosynthesis.

### 3.3. Generation of a Deletion Mutant

**Figure 3:**
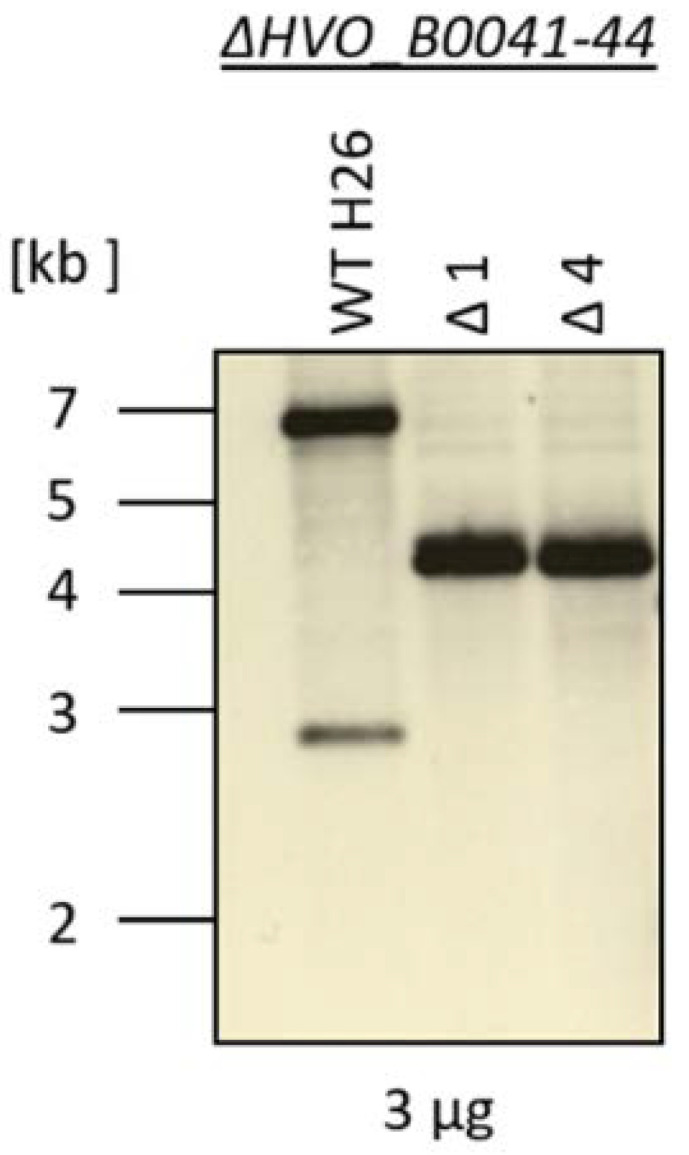
Southern blot analysis of the genomic DNA from the wild-type H26 and from two clones of the *iuc* gene deletion mutant. The size marker is schematically indicated to the left.

A quadruple deletion mutant of the four *iuc* genes (HVO_B0041 to HVO_B0044) was generated using the so-called pop-in/pop-out method [10,14]. The deleted region, as well as its genomic position, is indicated in Figure 1. The genomes of the wild type and two clones of the deletion mutants were characterized by Southern blot analysis (Figure 3). The sizes of the hybridizing bands perfectly fit to the expected sizes of 7010 nt for the wild type and 4214 nt for the deletion mutant. Notably, the larger wild-type band was totally absent from the two deletion mutant clones, showing that they were homozygous for the deletion. This is important, because *H. volcanii* is highly polyploid [20,21], and several examples of heterozygous strains, which contain wild-type and mutated genomes, were previously observed. The homozygosity of the clones was further underscored by multi-cycle PCR. These analyses showed that the deletion mutant was ideally suited for phenotypic comparisons with the wild type.

### 3.4. Fe-Dependent Growth of Wild Type and Deletion Mutant

**Figure 4:**
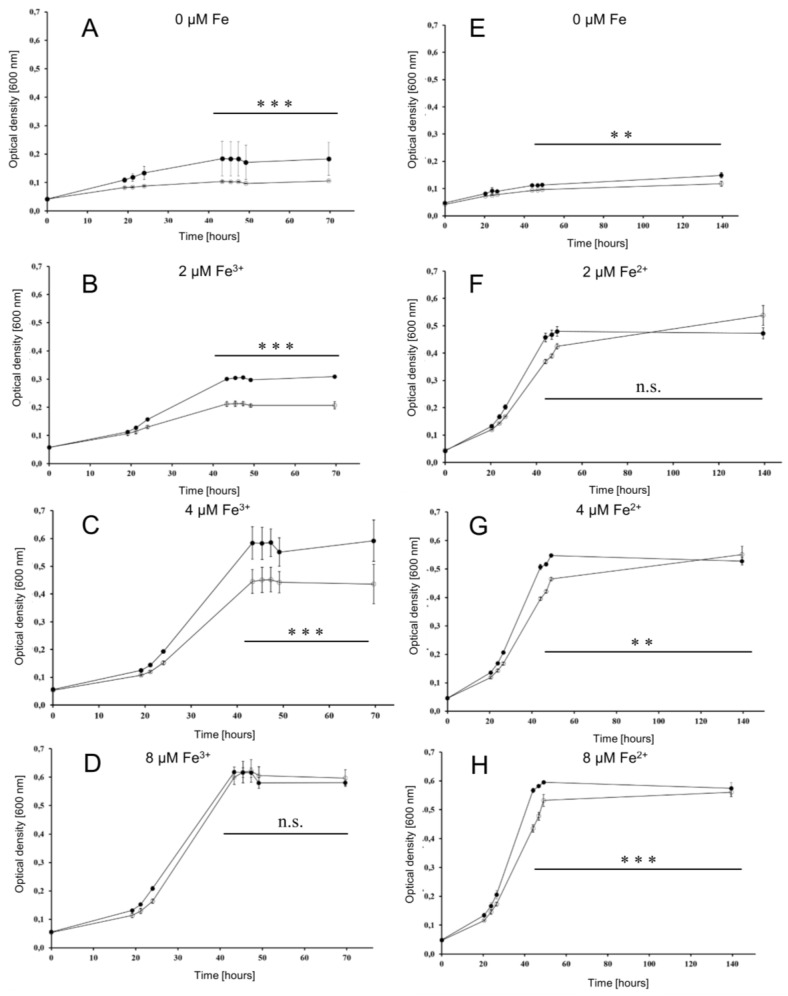
Growth curves of the wild type (filled symbols) and the *iuc* gene deletion mutant (open symbols) in the absence of iron and at three different iron concentrations, as indicated. Growth with Fe^3+^ is shown to the left (**A**–**D**), and growth with Fe^2+^ is shown to the right (**E**–**H**). Results of three biological replicates and their standard deviations are shown. The OD_600_ values at the last five time points were used to evaluate the significance of the difference between the wild type and *iuc* deletion mutant using an unpaired Student’s *t*-test (ns = non-significant; ** *p* < 0.01; *** *p* < 0.001).

**Figure 5:**
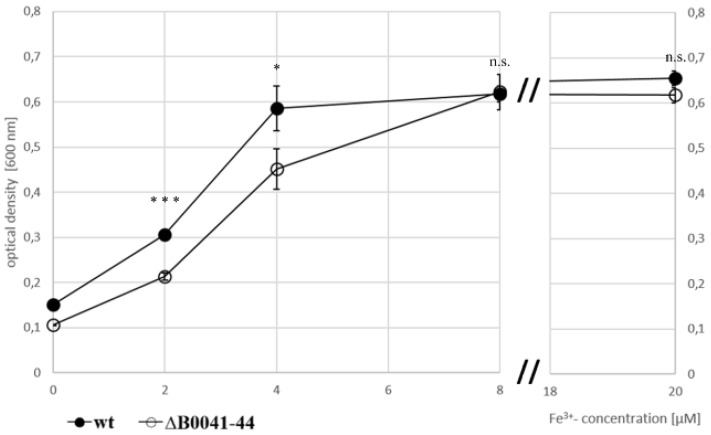
Growth yields of the wild type (filled symbols) and the *iuc* gene deletion mutant (open symbols) in the absence of iron and with four different Fe^3+^ concentrations, as indicated. Average values of three biological replicates and their standard deviations are shown. The growth yields were used to evaluate the significance of the difference between the wild type and *iuc* deletion mutant using an unpaired Student’s t-test (ns = non-significant; * *p* < 0.05; *** *p* < 0.001).

To investigate whether or not the iuc proteins are involved in iron acquisition at low iron concentrations, the wild type and the *iucABDC* deletion mutant were grown in the absence of iron and in the presence of 2 µM, 4 µM, and 8 µM FeCl_3_. The mutant had a clear growth disadvantage in the absence of FeCl_3_ (Figure 4A) and at the two lowest concentrations (Figure 4B,C), while, in contrast, the two strains grew indistinguishably at a concentration of 8 µM FeCl_3_ (Figure 4D). For a better visualization, the growth yields of the cultures were plotted against the FeCl_3_ concentrations, and the growth yields of cultures grown with 20 µM iron were added (Figure 5). Already at a concentration of 4 µM FeCl_3_, growth of the wild type was not iron-limited.

This was not true for the *iucABDC* mutant, which needs at least 8 µM FeCl_3_ to reach the full growth capability. The difference between the two strains was most prominent at low iron concentrations of 2 µM and 4 µM. These results clearly showed that the *iuc* gene products are important for full growth under very low FeCl_3_ concentrations. The lack of a phenotypic difference at concentrations of 8 µM or higher Fe^3+^ indicate that *H. volcanii* has a second Fe^3+^ importer, which is less efficient and siderophore-independent.

To elucidate whether the oxidation state of iron is important, the two strains were also grown at the identical concentrations of FeCl_2_ instead of FeCl_3_ (Figure 4E–H). At all Fe^2+^ concentrations, growth curves of the wild type and the *iuc* deletion mutant were indistinguishable, showing that the siderophore is specific for the oxidized state of Fe^3+^. Growth yields of wild type and mutant were at least as high with Fe^2+^ as with Fe^3+^, indicating that *H. volcanii* has several different iron importers, where one is siderophore-independent and specific for Fe^2+^. Growth yields with the very low concentration of 2 µM were much higher with Fe^2+^ than with Fe^3+^, which might reflect the higher solubility of Fe^2+^.

Taken together, the clear growth differences between the wild type and the *iuc* deletion mutant strongly indicated that *H. volcanii* synthesizes and exports an Fe^3+^-specific siderophore, which is important for iron uptake at low iron concentrations.

### 3.5. Verification of Siderophore Synthesis and Export

**Figure 6:**
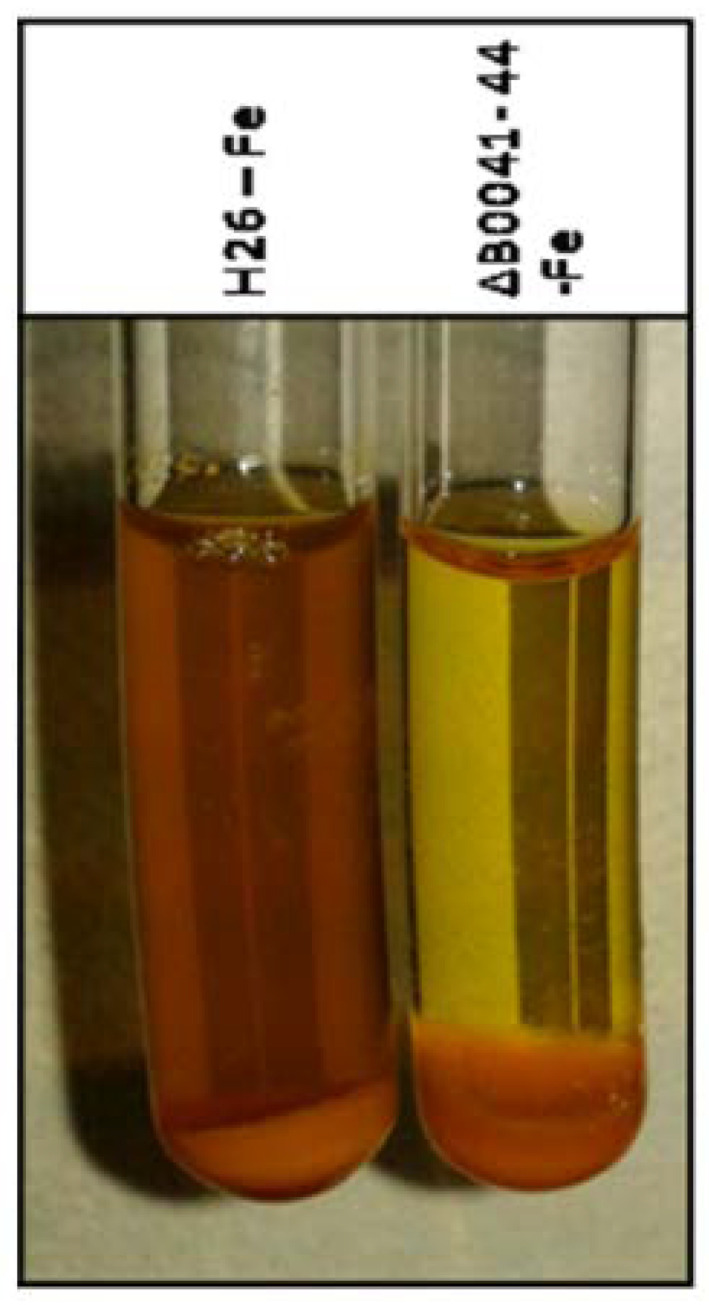
Experimental analysis of siderophore production and export. The wild type (left) and the *iuc* gene deletion mutant (right) were grown in the absence of iron in the medium. The cells were pelleted and FeCl_3_ was added to the supernatant. Development of a brown color indicates the presence of a Fe^3+^ siderophore in the culture supernatant.

For direct proof of siderophore production and export, cultures of the wild type and the *iuc* deletion mutant were grown for five days in the absence of iron. The cells were pelleted, and the supernatant was analyzed with the FeCl_3_ assay [16]. Figure 6 shows the presence of an Fe^3+^ siderophore in the supernatant of the wild-type culture (brown color), which is lacking in the supernatant of the mutant culture.

**Figure 7:**
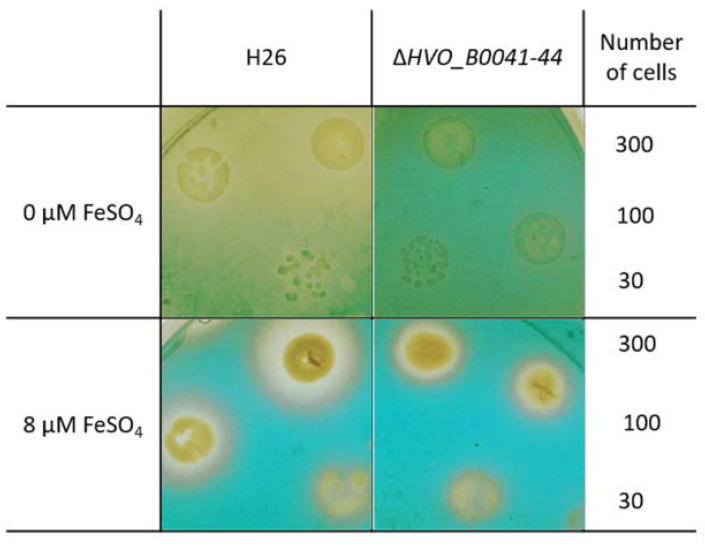
Overlay CAS (O-CAS) assay for the production and export of a Fe^3+^ siderophore. Tenfold dilutions of the cell cultures of the wild type (**left**) and the *iuc* gene deletion mutant (**right**) were spotted onto agar plates. The number of cells that were spotted is indicated in the rightmost column. The plates were either devoid of iron (**top**) or contained 8 µM iron (**bottom**). After four days of growth the plates were overlaid with a solution containing Fe^3+^ and the CAS reagent. Development of a blue-green color indicated the presence of free Fe^3+^, while retention of the yellow color of the medium indicates the lack of free iron, either because it was complexed by a Fe^3+^ siderophore (top left) or because it was taken up by the cells of the colonies (**bottom**).

The so-called O-CAS assay [17] was used as the second experimental proof for siderophore production and export. Cultures of the wild type and the *iuc* deletion mutant were grown overnight in the absence of iron. The cell density was determined by counting, and several dilutions with known numbers of cells were spotted on agar plates. After cultivation for four days, the plates were overlaid with the CAS solution, which contained 0.1 mM FeCl_3_ and the CAS reagent chromazurol. In the absence of a siderophore, a blue-green Fe^3+^–CAS complex develops. If a siderophore is present, it captures the Fe^3+^ such that color formation is suppressed. Figure 7 shows that, for cells grown in the absence of iron, the plate remained yellow around the wild-type colonies. In contrast, the whole plate became green-blue around the colonies of the *iuc* deletion mutant, indicating the absence of a siderophore. When the plates contained 8 µM FeCl_3_ during the incubation, small yellow halos were found around the colonies of both wild type and mutant, indicating Fe^3+^ depletion due to siderophore-independent uptake. The absence of halos around the mutant in the plates lacking iron indicated that the corresponding importer is not induced under iron starvation conditions.

Taken together, the two assays revealed that *H. volcanii* produces and exports an Fe^3+^-specific siderophore during iron starvation in batch culture, as well as during growth on solid media.

### 3.6. Bioinformatic Analysis of the Six Iuc Operon-Encoded Proteins

Selected characteristics of the four putative iuc proteins and the two adjacently encoded proteins HVO_B0045 and HVO_B0046 are listed in Table S2 (Supplementary Materials). They all are devoid of predicted transmembrane domains and signal peptides and, thus, can safely be assumed to be cytoplasmic proteins, as expected for siderophore biosynthesis enzymes. They all have the typical low isoelectric point of haloarchaeal proteins of about 4.5. Haloarchaea use the so-called “salt in” strategy for adaptation to their high salt environment, and they accumulate an equimolar salt concentration in their cytoplasm [22]. Their proteins have a high fraction of aspartate and glutamate, and a high charge density on the surface is important for competing with the salt for hydration. The amino-acid compositions of the six proteins HVO_B0041–HVO_B0046 and the proteome of *H. volcanii* are shown in Table S3 (Supplementary Materials). The fractions of aspartate and glutamate of the six proteins are even 1–2% higher than that of the proteome. Overall, the amino-acid compositions of all six proteins are very similar to that of the proteome, with very slight differences, e.g., the four Iuc proteins have slightly increased tyrosine fractions, and HVO_B0046 has a very low tryptophan content.

### 3.7. Phylogenetic Distribution of Iuc and Related Genes, Putative Enzyme Activities, and Proposal of a Siderophore Biosynthesis Pathway

To elucidate the phylogenetic distribution of the *H. volcanii* siderophore in other species of archaea and in bacteria, the protein sequences of HVO_B0041–HVO_B0046 were used for BLASTp searches in the UniProt database. Separate searches were performed for the taxonomic subsets of “archaea” and “bacteria”, and the 1000 most similar proteins were retrieved. The results are summarized in Table S4 (Supplementary Materials). The occurrence of the four iuc proteins is restricted to haloarchaea, to less than 100 species (HVO_B0042, HVO_B0043) or about 200 species (HVO_B0041, HVO_B0044). The two proteins HVO_B0045 and HVO_B0046 are encoded in more than 1000 archaeal genomes of diverse groups of archaea and, therefore, seem to have an alternative role in addition to their participation in siderophore synthesis (see Section 4).

**Figure 8:**
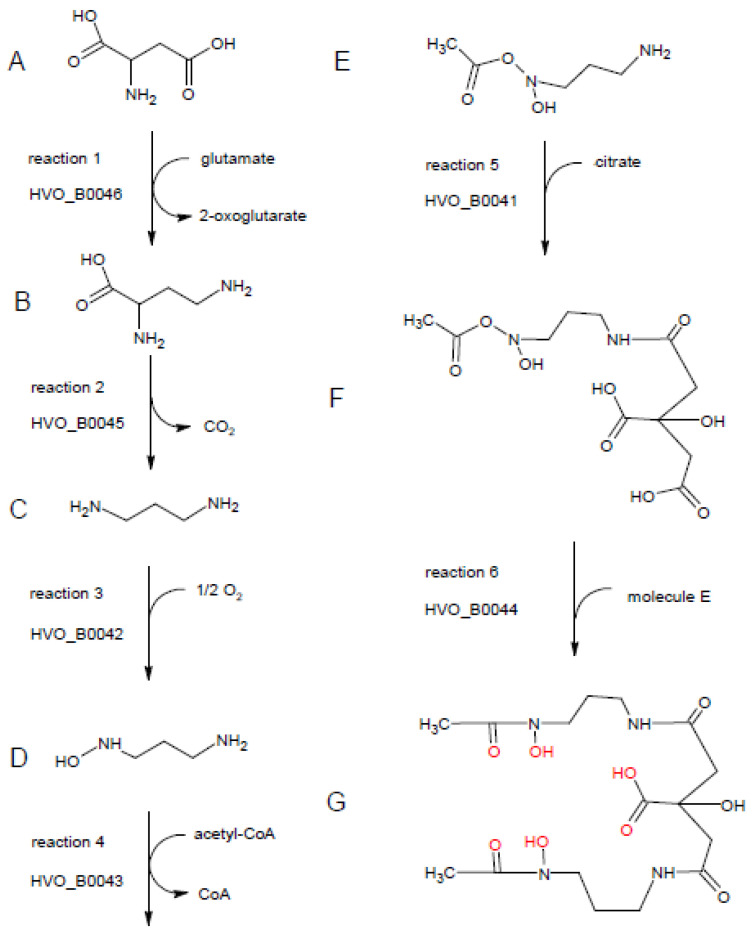
Putative enzymatic activities of HVO_B0041–HVO_B0046 and proposed biosynthetic pathway of Schizokinen. The substrate, intermediates, and the product are denoted with letters from A to G. The reactions are numbered and the corresponding enzyme designation is shown. For details, see text. The graphic was produced with ChemSketch (www.acdlabs.com).

Homologs of all six proteins are encoded in more than 1000 bacterial species. Therefore, the ability to produce this or a similar siderophore seems to be very widespread in several phylogenetic groups of bacteria. In all six cases, proteins from diverse genera of Bacillaceae (e.g., *Bacillus*, *Paenibacillus*, *Fictibacillus*, *Halobacillus*, *Brevibacillus*) were among the top scores in the list of the most similar proteins. Therefore, it seems that the six genes for the siderophore production in *H. volcanii* were obtained via lateral transfer from Bacillaceae species. Other bacterial groups that are prominent in the 1000 most similar homologs to all six proteins are *Streptomycetes* and *Cyanobacteria*. Therefore, in bacteria, the biosynthesis pathway is not restricted to one phylogenetic group, but occurs in very different groups. The most frequent annotations of the archaeal and, respectively, bacterial homologs of the six proteins are also listed in Table S4 (Supplementary Materials). Based on these annotations and the excellent annotation of the *H. volcanii* genome, putative enzymatic activities, as well as a siderophore biosynthesis pathway, are proposed. The complete biosynthesis pathway is shown in Figure 8, and the individual enzymatic steps are discussed in the following paragraphs.

The first reaction is proposed to be catalyzed by HVO_B0046. The most frequent annotations of the HVO_B0046 homologs are “diaminobutyrate pyruvate aminotransferase” and “diaminobutyrate oxoglutarate aminotransferase”, and the latter annotation was chosen for the *H. volcanii* genome. The name denotes the back-reaction of the siderophore biosynthesis pathway; in the forward reaction, it is proposed that the enzyme converts aspartate (molecule A in Figure 8) to 2,4-diaminobutyrate (molecule B), taking the amino group from glutamate, which is converted to 2-oxoglutarate. The choice for the C4 instead of the C3 substrate/product was taken based on the annotation of the next enzyme, HVO_B0045.

Most homologs of HVO_B0045, which is proposed to catalyze reaction 2, are annotated as decarboxylases, acting on one of several potential substrates. Here, 2,4-diaminobutyrate is the only diamino compound and, thus, should be the substrate of HVO_B0045, as also proposed in the genome annotation. The product after removal of the carboxylic group is 1,3-diaminopropane (molecule C).

Accordingly, 1,3-diaminopropane is proposed to be the substrate of HVO_B0042, which is a monooxygenase and hydroxylates one of the two amino groups, yielding 1-amino-3-(*N*-hydroxy)aminopropane (molecule D). The proposed annotation of “1,3-diaminopropane monooxygenase” is not found for any of the archaeal and bacterial homologs, while it is only found in the genome annotation of *H. volcanii* and other haloarchaeal genomes that were manually curated in detail [23].

The fourth proposed reaction is the acetylation of *N*-hydroxy-diaminopropane by HVO_B0043, yielding 1-amino-3-(*N*-hydroxy,*N*-acetyl)aminopropane (molecule E). The homologs carry very different annotations, including misleading ones like “iron transport protein”. Many are annotated as acetyltransferases, without an annotated substrate or with different substrates.

The fifth proposed biosynthesis step is the condensation of 1-amino-3-(*N*-hydroxy,*N*-acetyl)aminopropane with citrate. The step could be catalyzed by HVO_B0041 or HVO_B0044, which both could catalyze the condensation of citrate with another compound. Accordingly, many homologs of both proteins are annotated as “IucA/IucC family protein”. We propose that the reaction is catalyzed by HVO_B0041, because the bacterial species of the genus *Francisella* contain a fusion protein composed of the homolog of HVO_B0041 (N-terminal, about 600 amino acids) and the homolog of the acetyltransferase HVO_B0043 (C-terminal, about 200 amino acids). According to the “Rosetta Stone” principle, the non-fused homologs should also interact and perform subsequent reactions [24]. The product of reaction 5 is 1-(*N*-citryl)-3-(*N*-hydroxy,*N*-acetyl)diaminopropane (molecule F).

If that would be true, the sixth and last step would be catalyzed by HVO_B0044. It is the condensation of molecule F with a second molecule E to yield a symmetrical compound (molecule G) with three hydroxyl groups and three carbonyl groups, which are in perfect configuration to bind one Fe^3+^ ion (highlighted in red in Figure 8). This compound (molecule G) was first discovered in culture supernatants of *Bacillus megaterium* and named Schizokinen, because cells did not divide in its absence; thus, it was erroneously thought to be a cell division factor [25]. Later, the structure of Schizokinen was determined, and it was found to be an Fe^3+^ siderophore [26]. In the absence of iron, cells do not grow and, thus, do not divide, which explains the initial observation.

Taken together, the discovery that the genes HVO_B0041–HVO_B0046 are transcribed into one hexacistronic operon, which is essential for siderophore production in *H. volcanii*, led to the assumption that all six encoded proteins are involved in siderophore biosynthesis. Based on bioinformatic analyses, using the annotations of bacterial and archaeal homologs and bacterial gene fusions, a proposal for the complete biosynthesis could be developed, which starts with aspartate, uses glutamate, O_2_, and acetyl-CoA as group donors, incorporates citrate, and yields the hyroxamate siderophore Schizokinen.

## 4. Discussion

There are only very few studies on siderophores of archaea (only 20 entries in PubMed with “siderophore AND archae*”), in contrast to siderophores of bacteria (2128 entries; accessed on 28 April 2020). Our results unequivocally show that the *iuc* genes of *H. volcanii* are essential for the production of an Fe^3+^ siderophore. The genes are transcribed into a hexacistronic transcript, strongly indicating that the neighboring genes HVO_B0045 and HVO_B0046 also belong to the siderophore biosynthesis gene cluster.

It is remarkable that the six genes are transcribed into only one single transcript of about 9000 nt. In *H. volcanii*, several cases were reported where gene clusters are transcribed into several transcripts of different lengths, due to only partial termination after proximal genes and/or the presence of additional promoters within the gene clusters [12,27]. Furthermore, for *Halobacterium*, a high prevalence for operon-internal promoters was also reported [28]. In light of these results, the presence of only one single hexacistronic transcript seems to be a very strong indication that all six proteins are needed simultaneously and that they are part of one biological pathway.

Five of the six genes overlap by 4 nt, and the distance between the remaining gene pair is 8 nt, which is too small to allow independent translation initiation, indicating that translational coupling occurs throughout the whole transcript. Recently, it was shown that translational coupling at overlapping genes in *H. volcanii* is very effective, and that de novo translation initiation at downstream genes does not occur at all [19]. If the coupling efficiency at the hexacistronic *iuc* transcript is also very high, this would result in about equimolar amounts of the six enzymes. In addition, it was shown that co-translational association of subunits to heteromeric protein complexes needs the presence of the respective gene on one transcript in *Escherichia coli* [29]. Therefore, it seems possible that two or more of the six enzymes form heteromeric complexes, which could enhance the efficiency of the biosynthetic pathway due to substrate shuttling. To our knowledge, this possibility is not yet investigated in biosynthesis pathways of any siderophore in any species.

The expression analysis gave a first indication that the *iuc* gene cluster is involved in iron metabolism. The hexacistronic transcript is hardly detectable at the “high” concentration of 40 µM Fe^3+^, while it is tremendously upregulated under iron starvation. Such a high degree of differential expression is typical for siderophore biosynthesis genes, because, on the one hand, iron is an essential element for cell growth and survival, while, on the other hand, high iron concentrations are toxic to the cell. Under oxic conditions, a surplus of iron leads to the formation of reactive oxygen species (ROS) and, thus, oxidative stress and damage [30].

The generation and characterization of the quadruple *iuc* deletion mutant gave final proof that the gene cluster is essential for the biosynthesis of an iron-specific siderophore (Figure 4, Figure 5, Figure 6 and Figure 7). The deletion mutant had a considerable growth defect at very low concentrations of Fe^3+^, but not at the same concentrations of Fe^2+^ (Figure 4). This result proves that the siderophore is specific for the oxidized form of iron, which is typical for iron siderophores and is in congruence with the much lower solubility of Fe^3+^, compared to Fe^2+^. It is widely believed that Fe^2+^ is rapidly oxidized to Fe^3+^ under aerobic conditions. However, a quantitative analysis of the iron oxidation kinetics by oxygen in aqueous solutions showed that the oxidation rate is highly influenced by various factors [31]. For example, Fe^2+^ oxidation is 1000-fold slower at pH 6 than at pH 9, and it is 10-fold slower in seawater than in distilled water. It might even be considerably slower in the high-salt medium of *H. volcanii* than in seawater; however, of course, quantitative values for high-salt media are not available. In any case, the growth curve differences between the Fe^3+^ and Fe^2+^ cultures show that Fe^2+^ was not oxidized during the course of the experiment.

The total absence of an Fe^3+^ siderophore in the supernatant of the deletion mutant showed that the *iuc* gene cluster is the only siderophore biosynthesis gene cluster in *H. volcanii*. This is notable, because a variety of genes experienced considerable expansions in gene number in haloarchaea, e.g., genes for the basal transcription factors TBP (TATA box binding protein) and TFB (Transcription factor B), for the replication protein ORC (Origin Recognition Protein), for DNA repair proteins MutS/L, etc. [32]. In contrast to *H. volcanii*, many bacteria produce more than one siderophore [3,4].

The search for homologs in archaea revealed that the entire cluster of six genes is restricted to haloarchaea. Two of the six genes, HVO_B0045 and HVO_B0046, are also present in a high number of other archaea and, thus, they must have a function separate from siderophore biosynthesis (discussed below). Homologs of all six genes of the *iuc* gene cluster are present in a very high number of bacteria of several phylogenetic groups, indicating that haloarchaea obtained the gene cluster via lateral transfer. For all six proteins, homologs from different genera of Bacillaceae scored very high in the lists of the 1000 most similar proteins. Therefore, it seems very likely that haloarchaea obtained the gene cluster via lateral transfer from a species of this phylogenetic group. Notably, Bacillaceae include halophilic species that might coexist with halophilic archaea in the same environments, e.g., *Bacillus halophilus, B. salexigens*, and *B. natronophilus*, as well as members of the genus *Halobacillus* and *Oceanobacillus* [33,34,35,36].

Bioinformatic analyses of the annotation of the homologs of all six proteins HVO_B0041–HVO_B0046 led to the prediction that they are involved in the synthesis of the siderophore Schizokinen (Figure 8). The analyses underscored the annotation of the *H. volcanii* genome, which is of especially high quality due to the extensive manual curation [23]. Only IucA and IucC did not have an annotation of a specific enzymatic reaction, and HVO_B0046 was named according to the back-reaction (as in many homologs). Schizokinen was first described as a factor that is present in culture supernatants of *B. megaterium* that is important for the onset of growth after inoculation of synthetic medium and, thus, it was thought to be a growth factor [25]. Later, it was found that Schizokinen is not a growth factor, but an “iron transport compound”, and the structure of Schizokinen was determined [26]. Schizokinen is also produced by the halophilic species *B. halodurans* [5], which might co-exist with *H. volcanii*. Schizokinen is not only produced by *Bacillus* species, but also by some species of other phylogenetic groups of bacteria, e.g., *Rhizobia* and cyanobacteria [4,37].

Schizokinen binds Fe^3+^ with extremely high affinity (K_D_ = 10^−36^ M [38]). It is a siderophore of the hydroxamate type, where citrate is symmetrically modified with two molecules of 1-amino-3-(*N*-hydroxy-*N*-acetyl)aminopropane (Figure 8). Several structurally similar siderophores exist, e.g., aerobactin, rhizobactin 1021, and petrobactin [39,40]. Therefore, the lists of the 1000 most similar bacterial proteins to the proteins HVO_B0041–HVO_B0046, which were generated by BLAST searches, most probably not only contain enzymes involved in Schizokinen biosynthesis, but also enzymes involved in the biosynthesis of one of the structurally related siderophores. In any case, the presence of homologs in more than 1000 bacterial species underscores the widespread distribution of hydroxamate siderophores in bacteria of diverse phylogenetic groups.

The gene cluster HVO_B0041–HVO_B0046 is not only present in *H. volcanii*, but also in additional haloarchaea, e.g., species of the genera *Halobacterium*, *Haloterrigena*, *Haloorientalis*, and *Natrinema*. A global analysis of the synteny of the gene cluster in haloarchaea was not possible, because the SyntTax synteny analysis tool [41], which was proven to be very useful for previous analyses (e.g., Reference [42]), is restricted to the major chromosomes and, thus, is blind for genes encoded on minor chromosomes or plasmids. It is remarkable that many haloarchaea contain a siderophore biosynthesis pathway that starts with aspartate and uses glutamate as amino group donor, because the proteomes of halophilic archaea are highly enriched in these two amino acids, in comparison with the proteomes of non-halophilic species. A high negative charge density on the surface of halophilic proteins is important for their solubility in the high-salt conditions of the cytoplasm [22]. Therefore, haloarchaea have high capacities for the biosynthesis, as well as for the import, of aspartate and glutamate [43], which makes these amino acids ideal substrates for alternative pathways like siderophore biosynthesis.

The bioinformatic analyses showed that hydroxamate siderophore biosynthesis in archaea is confined to haloarchaea, and it is absent in other groups. However, the two proteins HVO_B0045 and HVO_B0046 are present in many more archaeal genomes of various groups than the four iuc proteins; therefore, they should have an alternative function to siderophore biosynthesis. The two enzymes convert aspartate into 1,3-diaminopropane, a polyamine. Polyamines have many important roles in cellular biology [44]. The presence of agmatine, spermidine, and branched-chain polyamines in archaeal species was reported, but not the presence of 1,3-diaminopropane [44]. It seems worth looking for the presence of 1,3-diaminopropane in archaeal species that contain solely HVO_B0045 and HVO_B0046, but not the *iuc* genes.

The six proteins encoded on the *iuc* gene cluster are essential and sufficient for the biosynthesis of the *H. volcanii* Fe^3+^ siderophore, most probably Schizokinen. However, further proteins are necessary for iron acquisition, i.e., the siderophore has to be exported from the cell, the Fe^3+^ siderophore complex has to be imported, and the Fe^3+^ has to be reduced to enable its removal from the siderophore complex. At present, the genes encoding all these functions in *H. volcanii* are unknown. On the 3′ side of the gene cluster, HVO_B0047 encodes a periplasmic binding protein of an ABC transporter with annotated iron specificity, which might be involved in Fe^3+^ siderophore complex uptake. However, the other subunit of the ABC transporter is not encoded near the *iuc* gene cluster. The *H. volcanii* genome contains a high number of ABC transporter genes, such that specific candidates are not obvious. Future experiments will be necessary to identify and characterize further proteins that are essential for iron acquisition via the siderophore that is synthesized by the enzymes encoded by the *iuc* gene cluster.

## 5. Conclusions

This study unequivocally revealed that the HVO_B0041–HVO_B0046 genes encode enzymes that are responsible for the synthesis of the only Fe^3+^ siderophore that is produced by *H. volcanii*. The six genes are transcribed into a single hexacistronic transcript, which is highly induced under iron starvation conditions. Two assays showed that a quadruple *iuc* deletion mutant was unable to produce and export a siderophore, in contrast to the wild type, and the mutant had a growth deficit at very low Fe^3+^ concentrations. Bioinformatic analyses of the protein sequences led to the proposal that *H. volcanii* produces the siderophore Schizokinen. In summary, this study represents the most in-depth analysis of siderophore biosynthesis in archaea, using genetic, molecular, and bioinformatic approaches.

## Supplementary Materials

The following are available online at https://www.mdpi.com/2218-273X/10/7/1072/s1: Table S1. List of oligonucleotides; Table S2. Selected features of the proteins HVO_B0041–HVO_B0046; Table S3. Amino-acid compositions of the H. volcanii proteome and of the proteins HVO_B0041–HVO_B0046; Table S4. Summary of BLAST searches with the proteins HVO_B0041–HVO_B0046; UniProtKB taxonomic subsets of archaea and bacteria.
