# Supplementary material for: Regulated Iron Siderophore Production of the Halophilic Archaeon Haloferax volcanii
Source: Biomolecules. 2020 Jul 17;10(7):1072. doi: 10.3390/biom10071072 (PMC7407949; doi:10.3390/biom10071072)
Supplement: Supplementary file 1 [file biomolecules-10-01072-s001.zip › biomolecules-819158-supplementary.pdf]

Supplementary Tables

Supplementary Table S1. List of primers

Name	Sequence
HVO_B0041-44 P1	CCCCGTGCGCGACGACGACG
HVO_B0041-44 P2	TCAGTCGTCCGCCGTGTCGTTCGGATTCATGCGTGTCGGTCG
HVO_B0041-44 P3	ATGAATCCGAACGACACGGCGGACGACTGACCCGCTGAG
HVO_B0041-44 P4	CGGTGGACTGGCCGGCCGC
B0044 S1 rev	AGCCACAGCACCGACA
B0044 S1 for	GCACCTCGGCCGAATA

Supplementary Table S2. Selected features of the proteins HVO_B0041 – HVO_B0046.

Protein name	Dat	Bdb	IucA	IucB	IucD	IucC
H. volcanii	HVO_B0046	HVO_B0045	HVO_B0044	HVO_B0043	HVO_B0042	HVO_B0041
UniProt ID	D4GP48	D4GP47	D4GP46	D4GP45	D4GP44	D4GP43
No. aa	459	521	593	213	457	612
mw	49.13	56,11	65.68	23.92	52.01	70.10
pI	4.6	4.4	4.5	4.9	4.5	4.6
Transmembr.	No	No	no	No	No	No
Signal pept.	No	No	no	No	No	No
RR..LAGC	No	No	no	No	No	No
domain	11-452	78-433	6-344	34-197	145-393	6-344
domain	IPR004637	IPR002129	IPRO025700	IPR000182	IPR007310	IPR025700
domain	2,4-diaminobutyrate 4-transaminase	Pyridoxalphosphate-dependend decarboxylase	L-lysine-6-monooxygenase	Acetyl-transferase (GNAT domain)	Aerobactin biosynthesis	Lys/Orn-oxygenase
Remark						

Supplementary Table S3. Comparison of the amino acid compositions of the four Iuc proteins with that of the proteome of *H. volcanii*. Considerable differences between the compositions of the three proteins and the average proteome are marked in blue (much lower fraction) and in red (much higher fraction) and are shown in bold.

Amino acid	*H. volcanii* Proteome	Dat	Bdb	IucA	IucB	IucD	IucC
	HVO_	B0046	B0045	B0044	B0043	B0042	B0041
	Fraction [%]	Fraction [%]	Fraction [%]	Fraction [%]	Fraction [%]	Fraction [%]	Fraction [%]
A	11.0	12,2	13,4	8.1	13.6	7.9	**6.2**
C	0.7	1,3	**1,5**	0.8	**1.4**	0.7	**0.3**
D	8.4	9,2	12,5	8.1	10.3	9.8	10.8
E	8.0	8,5	6,0	9.1	7.5	9.4	9.0
F	3.5	2,4	3,1	3.7	3.3	5.0	4.6
G	8.5	10,0	7,5	6.9	5.2	7.4	7.4
H	2.0	3,7	2,1	2.2	**4.2**	3.1	3.6
I	3.8	2,8	2,3	**1.7**	2.8	2.4	2.8
K	2.0	**1,1**	1,7	**1.2**	2.3	**1.1**	2.5
L	9.1	9,2	10,6	11.6	8.5	9.0	10.9
M	1.8	2,4	1,3	**0.7**	1.9	**0.9**	1.1
N	2.4	1,5	2,5	3.4	1.4	2.4	2.0
P	4.6	6,1	5,4	6.7	6.6	6.1	5.6
Q	2.4	1,5	1,9	2.9	1.4	3.1	2.3

R	6.7	7,2	7,7	7.1	8.5	8.1	7.7
S	5.9	3,9	4,6	6.1	**1.9**	3.5	4.4
T	6.2	5,2	5,6	5.6	6.6	5.3	5.4
V	9.2	8,9	7,7	9.3	7.0	8.5	7.2
W	1.1	**0,4**	0,8	0.7	1.4	1.5	1.8
Y	2.7	2,4	1,9	**4.2**	**4.2**	**4.8**	**4.6**
No. aa	1.168.832	459	521	593	213	457	612

Supplementary Table S4. Results of BLAST searches with the protein sequences of HVO_B0041 – HVO_B0046 in the UniProt taxonomic groups "Bacteria" and "Archaea", respectively. The 1000 best results were listed. The Table gives an overview of the number of species with homologs, the phylogenetic groups of these species, and the annotations of the homologs.

If 1000 homologs were retreaved, the p-value of Nr. 1000 is listed in parenthesis.

"Bacillus genera" summarizes many genera with Bacillus in their name, e.g. Paenibacillus, Halobacillus, Fictibacillus, Brevibacillus ets.

Protein	Archaea			Bacteria		
	No. species with homologs (p-value)	Phylogenetic groups	Annotations	No. species With Homologs (p-value)	Phylogenetic groups	Annotations
HVO_B0046	>1000<br>(2.6 E-57)	Haloarchaea<br>Methanogenic A.<br>div. other groups	Diaminobutyrate pyruvate aminotransf.<br>Diaminobutyrate Oxoglutarate aminotr.<br>Aspartate aminotransf.	>1000<br>(0.0)	Bacillus genera e.g. Paenib. etc.<br>Pseudomonas<br>Cyanobacteria<br>Streptomyces	Diaminobutyrate Pyruvate aminotransf.<br>Diaminobutyrate Oxoglutarate aminotr.<br>Aspartate aminotransf.
HVO_B0045	>1000<br>(5.0 E-10)<br>Including paralogs	Haloarchaea<br>Methanogenic A.<br>div. other groups	2,4-Diaminobutyrate decarboxylase<br>Glutamate decarboxylase<br>Aspartate decarboxylase	>1000<br>(5.6 E-111)	Bacillus genera<br>Enterobacteriaceae	2,4-Diaminobutyrate decarboxylase<br>Aspartate aminotransferase family

	No. species with homologs (p-value)	Phylogenetic groups	Annotations	No. species With Homologs (p-value)	Phylogenetic groups	Annotations
HVO_B0044	About 200	Only Haloarchaea	Iron transport protein<br>Siderophore biosynthesis pr.<br>IucA/IucC family protein	>1000<br>(2.5 E-118)	Bacillus genera<br>Streptomyces<br>Cyanobacteria	Iron transport protein<br>Petrobactin biosynthesis pr.<br>IucA/IucC family protein
HVO_B0043	87	Only Haloarchaea	Iron transport protein<br>Siderophore biosynthesis pr.	>1000<br>(1.3 E-22)	Bacillus genera<br>Streptomyces<br>Cyanobacteria	Acetyltransferase<br>Lysine acetyltransferase<br>Hydrocylysine acetyltr.<br>Rhizobactin biosynthesis pr.
HVO_B0042	85	Only Haloarchaea	Lysine monooxygenase<br>Monooxygenase	>1000<br>(1.4 E-116)	Bacillus genera<br>Streptomyces<br>Cyanobacteria	Lysine monooxygenase<br>Siderophore biosynthesis p.<br>Rhizobactin biosynthesis p.<br>Alcaligin biosynthesis p.<br>Lysine hydroxylase
HVO_B0041	About 200	Only Haloarchaea	Iron transport protein<br>IucA/IucC family protein	>1000<br>(5.0 E-116)	Bacillus genera<br>Streptomyces<br>Cyanobacteria	Iron transport protein<br>IucA/IucC family protein<br>Aerobactin synthase
